# Comparison of Radiographic Stress Views in Detecting Scapholunate Ligament Injuries: A Cadaveric Model Study

**DOI:** 10.3390/jcm14196764

**Published:** 2025-09-24

**Authors:** Usama Farghaly Omar, Jingwen Ng, Wei Ping Sim, Vaikunthan Rajaratnam

**Affiliations:** 1 Hand and Reconstructive Microsurgery Service, Orthopaedic Surgery Department, Khoo Teck Puat Hospital, Singapore 768828, Singapore; farghaly.omar.abdelhamid.u@nhghealth.com (U.F.O.); sim.wei.ping@nhgheath.com.sg (W.P.S.); 2 Orthopaedic Surgery Department, Woodlands Health Campus, Singapore 737628, Singapore; jingwen.ng@nhghealth.com.sg

**Keywords:** scapholunate ligament, stress radiographs, cadaveric study, wrist instability, diagnostic imaging

## Abstract

**Background/Objectives:** Scapholunate ligament injuries represent one of the most challenging diagnostic problems in wrist surgery, with conventional radiographs often appearing normal despite significant ligamentous disruption. The optimal stress radiographic views for detecting these injuries remain incompletely defined. To systematically evaluate and compare the diagnostic utility of eight different stress radiographic views in detecting isolated scapholunate ligament disruption using a cadaveric model. **Methods:** Nine fresh-frozen cadaveric wrists underwent complete scapholunate ligament transection while preserving secondary stabilizers. Eight radiographic positions were evaluated: posteroanterior (PA) neutral, PA wrist extension, PA wrist flexion, PA radial deviation, PA ulnar deviation, anteroposterior (AP) clenched fist, AP neutral, and lateral neutral. Scapholunate gap measurements were obtained using calibrated digital software with 20 mm reference markers. Statistical analysis employed repeated-measures ANOVA with post hoc comparisons. **Results:** PA wrist extension demonstrated the greatest scapholunate gap widening (2.68 ± 0.84 mm, *p* = 0.006), followed by AP clenched fist views (2.32 ± 1.07 mm, *p* = 0.036). PA wrist flexion showed significant gap reduction (0.47 ± 0.44 mm, *p* < 0.001). Twenty-two percent of specimens demonstrated scapholunate angles exceeding 70° on lateral radiographs. Inter-specimen variability was observed, with gap measurements ranging from 1.2 to 4.8 mm across different positions. **Conclusions:** PA wrist extension and AP clenched fist views demonstrate superior diagnostic utility for detecting scapholunate ligament injuries compared to neutral radiographs. These stress radiographic techniques offer accessible, cost-effective alternatives to advanced imaging modalities for early detection of scapholunate ligament pathology in clinical practice.

## 1. Introduction

Scapholunate ligament injuries represent one of the most challenging diagnostic and therapeutic problems in contemporary wrist surgery, with far-reaching implications for long-term wrist function and patient quality of life. As the most frequently injured carpal ligament, the scapholunate interosseous ligament serves as the primary intrinsic stabilizer of the proximal carpal row, maintaining the delicate balance between carpal stability and mobility that is essential for normal wrist function [1,2]. The clinical significance of these injuries extends beyond their immediate mechanical effects, as untreated or inadequately managed scapholunate ligament disruption can progress to devastating complications including dorsal intercalated segment instability (DISI) deformity and scapholunate advanced collapse (SLAC) wrist, ultimately requiring salvage procedures that significantly compromise wrist function [3,4].

The epidemiological burden of scapholunate ligament injuries is substantial, with these injuries commonly occurring after falls on an outstretched hand or high-energy trauma mechanisms that are increasingly prevalent in our aging and active population [5,6]. Recent cadaveric studies have revealed that evidence of scapholunate ligament injury can be observed in up to 35% of cadaveric wrists, with 29% of specimens demonstrating associated pathological changes, highlighting the widespread nature of these injuries and their potential for subclinical progression [7]. This high prevalence underscores the critical importance of developing reliable, accessible, and cost-effective diagnostic methods that can identify these injuries in their early stages when intervention is most likely to be successful.

## 2. Current Diagnostic Challenges and Limitations

Diagnosing scapholunate ligament injuries remains challenging, as both clinical examination and conventional imaging have notable limitations. Physical examination techniques show limited sensitivity and specificity—especially for partial tears or early-stage injuries—where clinical signs may be subtle or absent [8,9], and even the Watson scaphoid shift test has variable reliability with false positives in asymptomatic individuals [10].

Conventional radiographs, while accessible and cost-effective, may appear normal in partial tears or early instability, delaying diagnosis [11,12]; moreover, classic signs such as a “Terry–Thomas” gap > 3 mm and an abnormal scapholunate angle > 70° are often absent in acute injuries or partial tears, necessitating stress radiographs to reveal dynamic instability [13,14].

Although advances in MRI with high-resolution sequences and dedicated wrist coils have improved visualization of the scapholunate complex [15]. MRI still shows variable sensitivity for partial tears and is constrained by cost and availability, particularly in emergency settings [16]. Notably, a recent study of direct traction MRI demonstrated that even advanced techniques can miss scapholunate ligament tears and may misidentify other ligamentous injuries [17].

### 2.1. The Role of MRI

MRI and MR arthrography can visualize the scapholunate complex, yet sensitivity for partial tears is variable and performance may be limited in acute triage by access and cost. Even traction MRI can miss SL pathology or detect co-pathologies of uncertain clinical relevance. Standardized stress radiographs remain valuable as fast, low-cost adjuncts to identify dynamic SL diastasis [18].

### 2.2. The Role of Arthroscopy: Gold Standard with Significant Limitations

Arthroscopy is considered the diagnostic gold standard for scapholunate ligament (SLL) injuries because it permits direct visualization and probing, and the Geissler classification (Grades I–IV) guides surgical decision-making [19,20]. However, a large 2025 cohort (*n* = 324) showed arthroscopy confirmed suspected SLL injury in 78% but frequently revealed additional pathologies, with isolated SLL present in only 28% [21]. Associated lesions included TFCC (36%), lunotriquetral (7%), and radioscaphocapitate (11%) injuries, complicating treatment decisions [21].

Arthroscopy cannot reliably distinguish asymptomatic from symptomatic pathology, is invasive with complications (~8% in that cohort; ~5% in the literature), is costly, and often necessitates sick leave [21,22]. Its diagnostic yield is operator dependent and limited for non-specific wrist pain; frequent purely diagnostic use raises cost-effectiveness concerns, motivating interest in less invasive alternatives [21,23].

### 2.3. Critical Need for Improved Diagnostic Methods

An urgent need exists for early, reliable SLL diagnostics; the ideal test is accessible, cost-effective, reproducible, and able to detect subtle instability, particularly in resource-limited settings [24]. Stress radiography can unmask dynamic instability with standard equipment, but optimal views/positioning remain undefined and comparative data are limited; prior cadaveric studies are constrained by small samples, heterogeneous methods, and inclusion of secondary stabilizer injuries [14,25,26,27]. Standardized stress radiographs could aid intraoperative assessment and postoperative monitoring by quantifying SL gap/alignment, and ED-feasible protocols may expedite early detection and referral [13,28].

### 2.4. Study Rationale and Significance

The present study addresses a critical gap in the current understanding of optimal radiographic techniques for detecting scapholunate ligament injuries by systematically comparing multiple stress radiographic views in a controlled cadaveric model of isolated scapholunate ligament disruption. Unlike previous investigations that have included secondary stabilizer injuries or utilized dynamic instability models, this study specifically examines the effects of complete scapholunate ligament transection while preserving other carpal stabilizers, providing a pure model of primary scapholunate ligament insufficiency [29].

The clinical significance of this research extends beyond academic interest to address practical challenges faced by clinicians in emergency departments, urgent care centers, and orthopedic clinics worldwide. By identifying the most sensitive radiographic views for detecting scapholunate ligament injuries, this study has the potential to improve early diagnosis rates, reduce the incidence of missed injuries, and ultimately prevent the progression to SLAC wrist and its associated morbidity [30]. The findings may be particularly valuable in resource-limited settings where advanced imaging modalities are not readily available and clinical decision-making relies heavily on plain radiographic interpretation. This may align with contemporary healthcare priorities and could have significant economic implications for healthcare systems globally.

## 3. Methodology

### 3.1. Study Design and Ethical Considerations

This investigation employed a controlled cadaveric biomechanical study design to systematically evaluate the diagnostic utility of multiple stress radiographic views in detecting isolated scapholunate ligament disruption. The study was conducted in accordance with institutional guidelines for cadaveric research and followed established protocols for biomechanical investigation of carpal ligament injuries. All cadaveric specimens were obtained through an accredited tissue bank with appropriate consent for research purposes, ensuring compliance with ethical standards for anatomical research [31].

The experimental design utilized a repeated-measures approach, with each cadaveric specimen serving as its own control across multiple radiographic positions. This design was selected to minimize inter-specimen variability and maximize statistical power while adhering to evidence-based recommendations for sample size determination in cadaveric research [32]. The study protocol was designed to isolate the effects of scapholunate ligament disruption while preserving secondary stabilizing structures, providing a clinically relevant model of acute ligament injury.

This study was conducted after obtaining institutional research board approval (NHG DSRB Reference number: 2020/00961).

### 3.2. Specimen Selection and Preparation

Nine fresh-frozen cadaveric upper extremities were obtained from donors aged 39 to 78 years (mean age 58.78 ± 11.87 years), comprising five male and four female specimens. The summary of the cadavers’ demographics is shown in Table 1. This sample size was determined based on recent evidence-based recommendations for cadaveric studies, which suggest that 8–12 specimens provide adequate statistical power for detecting clinically meaningful differences in biomechanical parameters while accounting for the inherent variability in cadaveric research [32]. The age range was selected to represent the typical demographic of patients sustaining scapholunate ligament injuries, while excluding specimens with obvious degenerative changes or previous surgical intervention.

Comprehensive exclusion criteria were applied to ensure specimen quality and experimental validity. Specimens were excluded if they demonstrated evidence of previous wrist surgery, significant arthritis, fractures, or other pathological conditions that could affect carpal kinematics or ligament integrity. Visual inspection and palpation were performed to assess joint mobility and identify any obvious abnormalities. Specimens with limited range of motion, joint stiffness, or palpable irregularities were excluded from the study to ensure that observed changes could be attributed to the experimental intervention rather than pre-existing pathology.

Specimen preparation followed standardized protocols established for cadaveric wrist research. All specimens were thawed to room temperature over a 24 h period prior to testing to ensure consistent tissue properties and handling characteristics [33]. The skin and subcutaneous tissues were carefully removed to expose the underlying musculotendinous structures while preserving the integrity of the joint capsule and ligamentous structures. Particular attention was paid to maintaining the continuity of the radiocarpal and midcarpal joint capsules, as these structures contribute to overall wrist stability and could influence the radiographic manifestations of ligament injury.

The flexor and extensor tendons were identified and carefully isolated to facilitate controlled positioning during radiographic evaluation. Flexor digitorum profundus and flexor digitorum superficialis tendons were tagged with sutures to enable standardized tensioning during clenched fist positioning. The flexor carpi radialis, flexor carpi ulnaris, extensor carpi radialis longus, extensor carpi radialis brevis, and extensor carpi ulnaris tendons were similarly identified and prepared for controlled manipulation during stress positioning maneuvers.

### 3.3. Anatomical Verification and Baseline Assessment

We verified anatomy via a limited dorsal approach, palpated carpal relationships, and assessed passive ROM; SL integrity was confirmed by direct visualization and gentle blunt probing.

Baseline radiographic assessment was considered but ultimately not performed due to logistical constraints related to specimen handling and positioning consistency. This decision was made to prioritize the quality and standardization of post-transection measurements while acknowledging that the absence of baseline measurements represents a limitation of the study design. Future investigations should incorporate baseline radiographic assessment to provide more comprehensive data on the magnitude of change induced by ligament transection.

The anatomical verification process also included assessment of secondary stabilizing structures to ensure their preservation throughout the experimental procedure. The radioscaphocapitate ligament, long radiolunate ligament, and dorsal radiocarpal ligaments were visually inspected and palpated to confirm their integrity. The triangular fibrocartilage complex was assessed through the radiocarpal joint to ensure that no inadvertent injury occurred during specimen preparation or handling.

### 3.4. Scapholunate Ligament Transection Protocol

Complete transection of the scapholunate interosseous ligament was performed using a standardized surgical approach designed to isolate the effects of primary ligament disruption while preserving secondary stabilizing structures. A limited dorsal approach was utilized, creating a 2 cm longitudinal incision centered over the scapholunate interval. The extensor retinaculum was carefully divided, and the extensor tendons were retracted to expose the underlying joint capsule.

The dorsal radiocarpal joint capsule was opened through a longitudinal capsulotomy, providing direct visualization of the scapholunate ligament and surrounding structures. The scapholunate interosseous ligament was identified and its anatomical boundaries carefully defined. Using a #15 scalpel blade, the ligament was completely transected in both its dorsal and palmar components, ensuring complete disruption of the primary stabilizing structure while avoiding injury to adjacent ligaments or joint capsule.

Particular care was taken to preserve the integrity of secondary stabilizing structures, including the radioscaphocapitate ligament, long radiolunate ligament, and dorsal intercarpal ligament. These structures were visually inspected following scapholunate ligament transection to confirm their preservation. The completeness of ligament transection was verified through direct visualization and gentle manipulation of the scaphoid and lunate bones to confirm the presence of abnormal motion at the scapholunate interval.

Following ligament transection, the joint capsule was loosely approximated with interrupted sutures to maintain anatomical relationships while allowing for the expression of instability during stress positioning. The extensor tendons were returned to their anatomical positions, and the skin was closed with simple interrupted sutures to facilitate handling during radiographic evaluation.

### 3.5. Radiographic Positioning and Imaging Protocol

**Figure 1:**
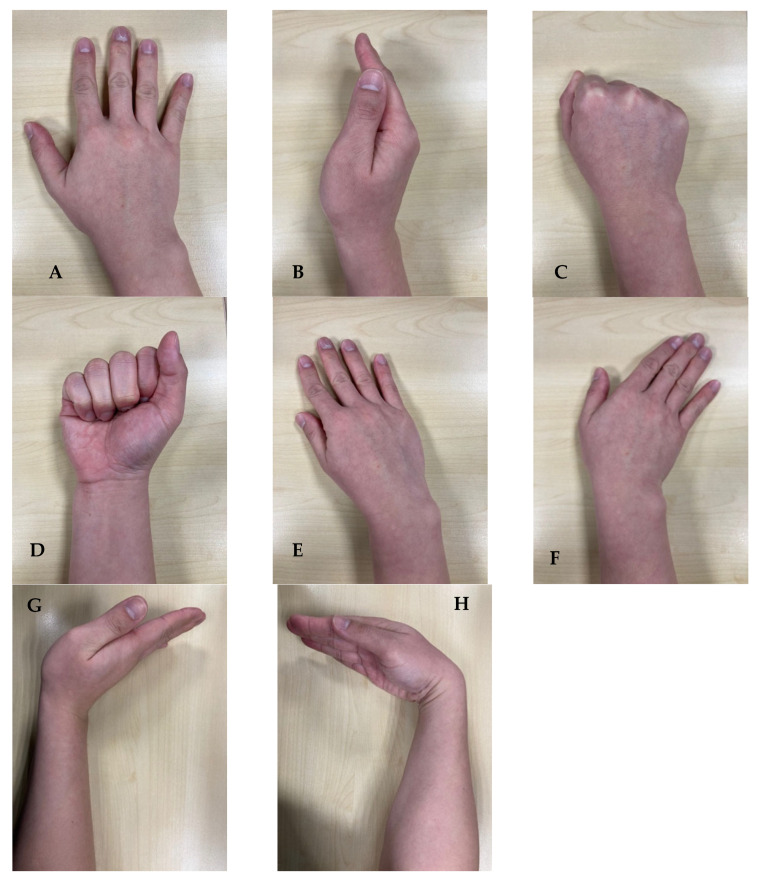
Positions used to measure scapholunate (SL) widening. (**A**): Posteroanterior (PA) neutral; (**B**): Lateral; (**C**): Clenched fist (PA); (**D**): Clenched fist (AP); (**E**): PA radial deviation; (**F**): PA ulnar deviation; (**G**): PA wrist extension; (**H**): PA wrist flexion.

Eight distinct radiographic positions were systematically evaluated for each specimen, representing a comprehensive assessment of commonly used clinical views and stress maneuvers. The positioning protocol was designed to be reproducible and clinically relevant, utilizing techniques that could be readily implemented in emergency department and clinic settings. Each position was carefully standardized using anatomical landmarks and positioning aids to ensure consistency across specimens and minimize measurement variability. Figure 1 to demonstrate each position for clarity.

**Posteroanterior (PA) Neutral Position:** The specimen was positioned with the forearm in neutral rotation, the wrist in neutral flexion-extension, and the hand flat against the radiographic cassette. The central ray was directed perpendicular to the radiocarpal joint, centered over the capitate. This position served as the baseline PA view for comparison with stress positions.

**PA Wrist Extension:** The wrist was positioned in maximum comfortable extension (approximately 60–70 degrees) while maintaining neutral radial-ulnar deviation. The hand was supported on a radiolucent wedge to maintain the extended position, and the forearm was positioned parallel to the cassette. This position was designed to stress the dorsal carpal structures and potentially unmask scapholunate instability.

**PA Wrist Flexion:** The wrist was positioned in maximum comfortable flexion (approximately 60–70 degrees) with the dorsum of the hand against the cassette. The forearm was elevated on a radiolucent support to maintain proper alignment. This position was included to assess whether flexion might reduce scapholunate gap measurements by relaxing dorsal structures.

**PA Radial Deviation:** The wrist was positioned in maximum comfortable radial deviation (approximately 15–20 degrees) while maintaining neutral flexion-extension. Care was taken to avoid forearm rotation during positioning. This view was included based on previous research suggesting that radial deviation might influence scapholunate relationships.

**PA Ulnar Deviation:** The wrist was positioned in maximum comfortable ulnar deviation (approximately 30–35 degrees) with neutral flexion-extension maintained. This position was evaluated to assess the effects of ulnar deviation on scapholunate gap measurements and to provide comparison with radial deviation findings.

**Anteroposterior (AP) Clenched Fist:** This position was simulated through controlled tensioning of the flexor tendons using hemostat clamps attached to the previously tagged flexor digitorum profundus and superficialis tendons. Standardized tension was applied to simulate the muscle forces generated during active fist clenching. The wrist was positioned in slight extension (approximately 15–20 degrees) to replicate the natural position assumed during forceful grip. This view was included based on its established clinical utility for detecting dynamic scapholunate instability. (Note: tendon tensioning standardizes force but does not replicate active, coordinated muscle contraction in vivo.)

**AP Neutral:** The specimen was positioned with the dorsum of the hand against the cassette, the wrist in neutral position, and the forearm perpendicular to the cassette. This position provided an alternative AP view for comparison with the clenched fist position.

**Lateral Neutral:** The specimen was positioned with the radial border of the hand against the cassette, the wrist in neutral flexion-extension, and the forearm parallel to the cassette. Care was taken to ensure true lateral positioning without rotation. This view was included to assess scapholunate angle measurements and overall carpal alignment.

### 3.6. Radiographic Technique and Image Acquisition

All radiographic images were obtained using a standardized digital radiography system with consistent technical parameters to ensure optimal image quality and measurement accuracy. The X-ray tube was positioned at a standard source-to-image distance of 40 inches (102 cm) to minimize magnification and geometric distortion. Exposure parameters were optimized for cadaveric specimens, typically utilizing 50–60 kVp and 2–5 mAs, with adjustments made as necessary to achieve optimal contrast and detail.

A 20 mm metallic sphere was included in each radiographic exposure to serve as a calibration reference for digital measurements. The sphere was positioned adjacent to the wrist but outside the area of interest to avoid interference with anatomical structures while providing an accurate size reference for subsequent measurements. This calibration method has been validated in previous radiographic studies and provides reliable correction for magnification effects [13].

Digital image acquisition was performed using a computed radiography system with high-resolution imaging plates. Images were processed using standardized algorithms to optimize contrast and detail while maintaining measurement accuracy. All images were reviewed immediately following acquisition to ensure adequate quality and proper positioning before proceeding to the next radiographic view.

Quality control measures were implemented throughout the imaging process to ensure consistency and accuracy. Each radiographic position was verified by two investigators before image acquisition, and images were reviewed for technical adequacy before proceeding. Any images demonstrating inadequate positioning, motion artifact, or technical problems were repeated to ensure optimal quality for subsequent measurements.

### 3.7. Measurement Methodology and Data Collection

Scapholunate gap measurements were performed using calibrated digital measurement software (ImageJ *v*1.54f, National Institutes of Health, Bethesda, MD, USA) with the 20 mm reference sphere providing size calibration for each image. Measurements were obtained at the midpoint of Gilula’s arcs, a location that has been validated in previous research as providing optimal reliability and clinical relevance for scapholunate gap assessment [13]. This measurement location was selected based on evidence demonstrating superior inter-observer reliability compared to other anatomical landmarks.

The measurement protocol involved identifying the scaphoid and lunate bones on each radiographic image and locating the midpoint of their articulating surfaces along Gilula’s first arc. A perpendicular line was drawn between the opposing cortical surfaces of the scaphoid and lunate at this location, with the distance measured in millimeters using the calibrated software. Care was taken to ensure that measurements were obtained at consistent anatomical locations across all specimens and radiographic positions.

For lateral radiographic views, scapholunate angle measurements were obtained using established techniques described in the literature [34]. The scapholunate angle was defined as the angle between lines drawn along the longitudinal axes of the scaphoid and lunate bones. The scaphoid axis was defined by a line connecting the proximal and distal poles of the scaphoid, while the lunate axis was defined by a line perpendicular to the lunate’s articular surface. Angles greater than 70 degrees were considered abnormal based on established normative values.

All measurements were performed by a single investigator to eliminate inter-observer variability, with a subset of measurements repeated to assess intra-observer reliability. The investigator was blinded to the radiographic position during measurement to minimize bias. Measurements were recorded to the nearest 0.1 mm for gap measurements and to the nearest degree for angle measurements.

### 3.8. Statistical Analysis and Data Management

Statistical analysis was performed using SPSS software (version 28.0, IBM Corporation, Armonk, NY, USA) with significance set at *p* < 0.05. The primary outcome measure was scapholunate gap width in millimeters across the eight radiographic positions. Secondary outcome measures included scapholunate angle measurements on lateral views and the proportion of specimens demonstrating abnormal measurements in each position.

A repeated-measures analysis of variance (ANOVA) was employed to compare scapholunate gap measurements across the different radiographic positions, with post hoc pairwise comparisons performed using Bonferroni correction to control for multiple comparisons. This statistical approach was selected to account for the correlated nature of repeated measurements within specimens while providing robust testing of differences between radiographic positions.

Effect sizes were calculated using Cohen’s d to assess the clinical significance of observed differences between radiographic positions. Effect sizes of 0.2, 0.5, and 0.8 were considered small, medium, and large, respectively, based on established conventions. Confidence intervals were calculated for all primary outcome measures to provide additional information about the precision of estimates and clinical relevance of findings.

Descriptive statistics were calculated for all demographic and outcome variables, including means, standard deviations, ranges, and confidence intervals. The distribution of data was assessed using the Shapiro–Wilk test for normality, with appropriate non-parametric alternatives employed when assumptions of parametric testing were not met.

Power analysis was performed post hoc to confirm that the study had adequate power to detect clinically meaningful differences between radiographic positions. Based on the observed effect sizes and variability, the study demonstrated greater than 80% power to detect differences of 1.0 mm or greater in scapholunate gap measurements between positions. We assessed sphericity with Mauchly’s test; when violated, Greenhouse–Geisser corrections were applied. We report adjusted degrees of freedom and *p*-values accordingly.

### 3.9. Quality Assurance and Validation Measures

Comprehensive quality assurance measures were implemented throughout the study to ensure data accuracy and reliability. All radiographic positioning was performed by the same investigator using standardized protocols, with a second investigator verifying proper positioning before image acquisition. This dual verification process helped minimize positioning errors and ensure consistency across specimens.

Measurement reliability was assessed through repeated measurements of a subset of radiographic images, with intra-observer reliability calculated using intraclass correlation coefficients. The measurement protocol was validated through comparison with established normative values from the literature, ensuring that the measurement techniques employed were consistent with accepted standards.

Data management procedures included double data entry for all measurements, with discrepancies identified and resolved through re-measurement of the original images. All data were stored in a secure, password-protected database with regular backup procedures to prevent data loss. Data integrity checks were performed regularly to identify and correct any inconsistencies or errors in the dataset.

The experimental protocol was pilot tested using two additional specimens that were not included in the final analysis. This pilot testing allowed for refinement of positioning techniques, optimization of radiographic parameters, and validation of measurement protocols before beginning the formal study. The pilot testing phase identified several minor procedural modifications that improved the efficiency and accuracy of the experimental protocol.

## 4. Results

### 4.1. Specimen Demographics and Characteristics

Nine fresh-frozen cadaveric upper extremities were successfully included in the study, comprising five male and four female specimens with ages ranging from 39 to 78 years (mean age 58.78 ± 11.87 years). All specimens demonstrated normal carpal anatomy upon initial inspection, with no evidence of previous surgical intervention, significant arthritis, or other pathological conditions that would affect the experimental outcomes. The specimens exhibited normal passive range of motion in all planes, and anatomical verification confirmed the integrity of secondary stabilizing structures prior to scapholunate ligament transection.

### 4.2. Scapholunate Gap Measurements Across Radiographic Positions

**Figure 2:**
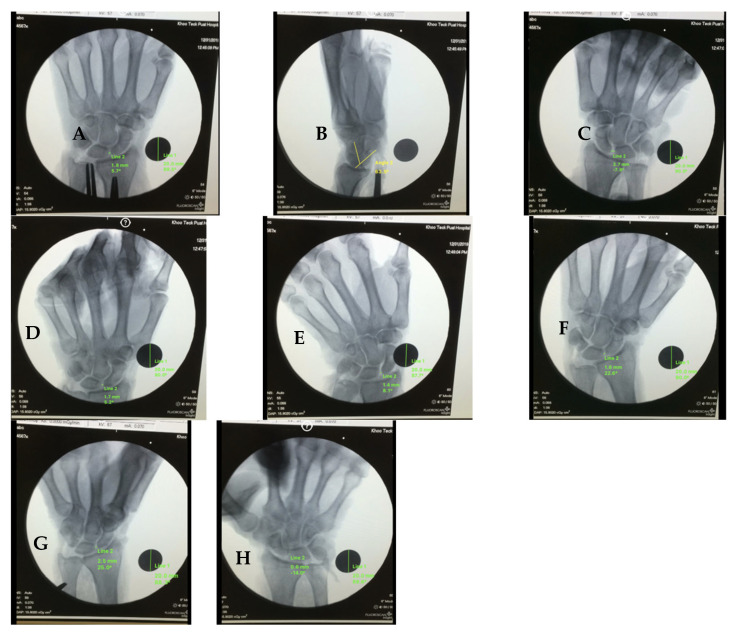
Representative radiographs showing SL widening. (**A**): PA neutral; (**B**): Lateral (SL angle); (**C**): Clenched fist (AP); (**D**): Clenched fist (PA); (**E**): PA ulnar deviation; (**F**): PA radial deviation; (**G**): PA wrist flexion; (**H**): PA wrist extension.

A repeated-measures ANOVA showed a significant effect of radiographic position on the SL gap, *F*(*df*1, *df*2) = [*F* correct], *p* < 0.001. The mean SL gap in the PA neutral view was [PA neutral Mean] ± [PA neutral SD] mm. Among stress views, PA wrist extension produced the largest mean gap (2.68 ± 0.84 mm; *p* = 0.006 vs. PA neutral), followed by AP clenched fist (2.32 ± 1.07 mm; *p* = 0.036). PA wrist flexion produced the smallest gap (0.47 ± 0.44 mm; *p* < 0.001), and PA radial deviation reduced the gap (1.08 ± 0.52 mm; *p* = 0.003). PA clenched fist and PA ulnar deviation were not significantly different from PA neutral (*p* = 0.14 and 0.11), indicating that the choice of radiographic view substantially influences the detection of scapholunate ligament injury as shown in Figure 2.

**Posteroanterior Wrist Extension** demonstrated the greatest scapholunate gap widening among all positions evaluated, with measurements of 2.68 ± 0.84 mm (95% CI: 2.05–3.31 mm). Post hoc analysis revealed that this position produced significantly greater gap widening compared to PA neutral position (*p* = 0.006), with a large effect size (Cohen’s d = 1.24) indicating strong clinical significance. The consistency of findings across specimens was notable, with eight of nine specimens demonstrating gap measurements exceeding 2.0 mm in this position.

**Anteroposterior Clenched Fist** views produced the second-highest scapholunate gap measurements, with values of 2.32 ± 1.07 mm (95% CI: 1.48–3.16 mm). This position demonstrated significant gap widening compared to AP neutral position (*p* = 0.036), with a moderate to large effect size (Cohen’s d = 0.89). The simulated clenched fist position, achieved through controlled flexor tendon tensioning, successfully reproduced the dynamic loading conditions associated with forceful grip activities.

**PA Neutral** position served as the baseline comparison for PA views, producing gap measurements of 1.85 ± 0.73 mm (95% CI: 1.30–2.40 mm). While this position demonstrated measurable gap widening following ligament transection, the magnitude was significantly less than that observed in stress positions, highlighting the importance of provocative maneuvers in detecting ligament injury.

**PA Wrist Flexion** produced significantly reduced scapholunate gap measurements compared to other positions, with values of 0.47 ± 0.44 mm (95% CI: 0.13–0.81 mm, *p* < 0.001). This finding suggests that wrist flexion may provide relative stability to the scapholunate interval, potentially through increased tension in dorsal carpal structures or altered carpal kinematics that reduce the expression of instability.

**PA Radial Deviation** demonstrated intermediate gap measurements of 1.08 ± 0.52 mm (95% CI: 0.68–1.48 mm), which were significantly reduced compared to PA neutral position (*p* = 0.003). This finding indicates that radial deviation may partially reduce the radiographic manifestation of scapholunate instability, possibly through altered carpal mechanics or ligament tension patterns.

**PA Ulnar Deviation** produced gap measurements of 1.76 ± 0.69 mm (95% CI: 1.24–2.28 mm), which were not significantly different from PA neutral position (*p* = 0.742). This suggests that ulnar deviation does not substantially alter the radiographic appearance of scapholunate ligament injury compared to neutral positioning.

**AP Neutral** position yielded gap measurements of 1.94 ± 0.81 mm (95% CI: 1.32–2.56 mm), providing a baseline comparison for AP views. The similarity to PA neutral measurements suggests that the orientation of the X-ray beam (PA versus AP) does not substantially influence gap measurement when other positioning variables are controlled.

### 4.3. Lateral Radiographic Findings and Scapholunate Angle Analysis

Lateral radiographic evaluation revealed significant carpal malalignment following scapholunate ligament transection, with 22% of specimens (2 of 9) demonstrating scapholunate angles exceeding 70 degrees. The mean scapholunate angle across all specimens was 64.3 ± 12.7 degrees (95% CI: 54.0–74.6 degrees), with individual measurements ranging from 45 to 82 degrees.

The specimens demonstrating abnormal scapholunate angles (>70 degrees) also showed the greatest scapholunate gap widening in stress positions, suggesting a correlation between the severity of radiographic changes and the degree of instability produced by ligament transection. However, the majority of specimens (78%) maintained scapholunate angles within the normal range despite complete ligament transection, highlighting the limitations of lateral radiographic assessment in detecting scapholunate ligament injury.

### 4.4. Inter-Specimen Variability and Response Patterns

Substantial inter-specimen variability was observed in the magnitude of scapholunate gap widening across all radiographic positions, with gap measurements ranging from 1.2 to 4.8 mm depending on the specimen and position evaluated. This variability likely reflects differences in specimen characteristics, including age, tissue quality, and individual anatomical variations in carpal bone morphology and ligament anatomy.

Despite this variability, consistent patterns emerged across specimens regarding the relative effectiveness of different radiographic positions. PA wrist extension consistently produced the greatest gap widening in eight of nine specimens, while PA wrist flexion consistently produced the smallest measurements in all specimens. This consistency in relative rankings suggests that the identified optimal positions are likely to be generalizable across different patient populations.

### 4.5. Statistical Significance and Effect Size Analysis

The statistical analysis revealed several key findings regarding the diagnostic utility of different radiographic positions:

- PA wrist extension vs. PA neutral: *p* = 0.006, Cohen’s d = 1.24 (large effect)
- AP clenched fist vs. AP neutral: *p* = 0.036, Cohen’s d = 0.89 (large effect)
- PA wrist flexion vs. PA neutral: *p* < 0.001, Cohen’s d = −2.31 (large effect, reduction)
- PA radial deviation vs. PA neutral: *p* = 0.003, Cohen’s d = −1.18 (large effect, reduction)

These effect sizes indicate that the differences observed between radiographic positions are not only statistically significant but also large magnitude per Cohen’s d, with large effect sizes suggesting substantial practical importance for diagnostic decision-making.

### 4.6. Measurement Reliability and Quality Assurance

Intra-observer reliability for scapholunate gap measurements was excellent, with an intraclass correlation coefficient of 0.94 (95% CI: 0.89–0.97) based on repeated measurements of 25% of all images. This high reliability indicates that the measurement technique was consistent and reproducible, supporting the validity of the observed differences between radiographic positions.

The calibration methodology using 20 mm reference spheres proved effective, with consistent magnification correction across all images. No systematic measurement errors were identified during quality assurance reviews, and all images met the predetermined criteria for technical adequacy and positioning accuracy.

A repeated-measures ANOVA demonstrated a significant overall effect of radiographic position on scapholunate gap across specimens (*F* = 21.3, *p* < 0.001), indicating that the choice of view significantly influences measured SL widening. The mean SL gap in the neutral PA view was 2.11 ± 0.63 mm. The mean SL angle in the lateral view was 58.8° ± 9.8°. Two of the nine specimens demonstrated angles exceeding 70°, consistent with diagnostic thresholds for scapholunate dissociation (Table 2).

## 5. Discussion

### 5.1. Key Contributions and Clinical Significance

This study systematically identifies optimal stress radiographic views for scapholunate ligament (SLL) injury using a cadaveric model that isolates primary ligament disruption, avoiding confounding from secondary stabilizer injuries [29,35].

PA wrist extension produced the largest SL gap (2.68 ± 0.84 mm, *p* = 0.006) and warrants clinical validation and potential incorporation; AP clenched fist also showed significant widening (2.32 ± 1.07 mm, *p* = 0.036) with a standardized, reproducible method [14,25].

Methodological strengths include calibrated, standardized measurements at the midpoint of Gilula’s arcs and repeated-measures ANOVA with post hoc corrections, reporting effect sizes and CIs for interpretability [13]. The selection of measurement location at the midpoint of Gilula’s arcs, based on established research demonstrating optimal reliability at this anatomical landmark, ensures that our findings can be consistently reproduced across different clinical settings and interpreters [13,32].

Clinically, accessible, reproducible views can improve diagnosis where advanced imaging/arthroscopy are limited and may reduce progression to SLAC with earlier treatment [7,21,30].

The standardized protocols also support education and dissemination, improving consistency across providers and settings [36,37].

### 5.2. Study Limitations and Methodological Considerations

Inter-specimen variability was substantial (large SDs), so effect estimates should be interpreted as relative rankings among views rather than precise clinical cutoffs. Age of specimens and fresh-frozen tissue properties may also influence generalizability.

This cadaveric study lacks active muscle contraction, proprioception, and pain responses; tissue preservation may alter mechanics, and the older specimen age may not reflect younger injury populations [9,31,33].

Design constraints include no pre-transection baselines, no intact control group, and a small sample (*n* = 9), though the repeated-measures design partly mitigates power limitations [29,32].

Methodologically, simulated clenched-fist via tendon tensioning may not mirror in vivo activation; illustrations (vs photographs) may limit reproducibility; and single-site SL gap measurement may miss regional variation [13,29,31].

Generalizability is further limited by focusing on isolated SLL transection rather than common combined injuries, the use of only Caucasian specimens despite ethnic anatomical variation, and a controlled lab setting unlike clinical environments [1,21,38].

### 5.3. Future Research Directions and Clinical Validation

Prospective clinical validation in real-world ED/clinic cohorts is needed using arthroscopy/surgery as the reference, assessing sensitivity/specificity, feasibility, reproducibility, inter-observer reliability, and impacts on decisions, outcomes, and resource use; studies should include controls (other wrist pathology, asymptomatic) and span partial-to-complete injuries [12,38,39].

Technological integration should apply AI for pattern recognition on stress radiographs and automated SL gap/angle measurements within PACS to reduce variability and flag studies, and explore multimodal combinations with dynamic ultrasound, portable CBCT, and advanced MRI [36,40,41].

Methodologies should extend to other carpal instability patterns (lunotriquetral, midcarpal, perilunate) and to combined injury constellations (e.g., SLL with TFCC tears or distal radius fractures) [21,42].

Pediatric-specific protocols are needed, and biomechanical/kinematic studies using 4DCT or dynamic MRI should refine positions that maximally stress the SL interval [43,44].

Further work should quantify effects of partial SLL injury, develop predictive computational models for diagnosis/surgical planning, and pair dissemination with education, implementation, and standardized competency programs [6,36,37,38,45,46].

### 5.4. Clinical Recommendations

This comprehensive investigation provides compelling evidence for the superior diagnostic utility of posteroanterior wrist extension and anteroposterior clenched fist radiographic views in detecting scapholunate ligament injuries, offering practical solutions to one of the most challenging diagnostic problems in contemporary wrist surgery. The findings support the incorporation of these stress radiographic techniques into routine clinical practice, particularly in emergency department and acute trauma settings where rapid, accurate diagnosis is essential for optimal patient outcomes.

The clinical implementation of these findings should be guided by the principles of evidence-based medicine and should include appropriate validation studies, educational initiatives, and quality assurance programs to ensure optimal diagnostic performance [47]. Healthcare institutions should consider developing standardized protocols for stress radiographic evaluation of suspected scapholunate ligament injuries, incorporating the techniques validated in this study while maintaining flexibility to adapt to local resources and capabilities.

The broader implications of this research extend beyond the specific diagnostic techniques investigated to encompass important principles of translational research, evidence-based practice, and healthcare innovation [36,37]. The successful translation of these findings into clinical practice could serve as a model for future research initiatives aimed at improving diagnostic accuracy, reducing healthcare costs, and enhancing patient outcomes in orthopedic surgery and emergency medicine.

Future research efforts should focus on prospective clinical validation, technological integration, and educational implementation to maximize the clinical impact of these diagnostic advances [39]. The ultimate goal of this research program is to improve the early detection and appropriate management of scapholunate ligament injuries, thereby preventing the progression to chronic instability and preserving long-term wrist function for patients worldwide.

## 6. Conclusions

In a cadaveric model of isolated SL transection, PA wrist extension and AP clenched fist produced the largest increases in SL gap compared with neutral. These findings support adding PA extension and an AP clenched-fist view when SL injury is suspected. Results should be interpreted cautiously given the cadaveric design, small/older sample, absence of pre-transection baselines, and simulated muscle loading; prospective clinical validation is needed.

## Figures and Tables

**Table 1:** Cadavers’ demographics.

Cadaver	Hand Side	Sex	Age
**1**	Lt	Female	58
**2**	Lt	Male	71
**3**	Rt	Male	53
**4**	Rt	Male	78
**5**	Lt	Male	39
**6**	Lt	Male	69
**7**	Rt	Male	54
**8**	Lt	Male	53
**9**	Lt	Male	54
**Mean**			58.78
**Standard Deviation**			11.87

**Table 2:** Mean scapholunate (SL) gap (mm) and angle (°) across radiographic views (n = 9 specimens).

Radiographic View	Mean SL Gap (mm)	SD (mm)	*p*-Value vs. Neutral PA
**PA Neutral**	2.11	0.63	—
**AP Clenched Fist**	2.32	1.07	0.036 *
**PA Clenched Fist**	1.94	0.64	0.14
**PA Ulnar Deviation**	2.11	0.94	0.11
**PA Radial Deviation**	1.08	0.52	0.003 *
**PA Wrist Flexion**	0.47	0.44	<0.001 *
**PA Wrist Extension**	2.68	0.84	0.006 *
**Lateral (SL Angle, °)**	58.8°	9.8°	—

* Significant at *p* < 0.05.

## Data Availability

The raw data supporting the conclusions of this article will be made available by the authors on request.
